# Diagnostic Fine-Needle Biopsy of Small Solid Pancreatic Lesions Using a Franseen Needle during Endoscopic Ultrasound Examination

**DOI:** 10.3390/diagnostics11010027

**Published:** 2020-12-25

**Authors:** Kosuke Takahashi, Ichiro Yasuda, Tatsuyuki Hanaoka, Yuka Hayashi, Yasuhiro Araki, Iori Motoo, Shinya Kajiura, Takayuki Ando, Haruka Fujinami, Kazuto Tajiri, Masami Minemura, Terumi Takahara

**Affiliations:** Third Department of Internal Medicine, University of Toyama, Toyama, 2630 Sugitani, Toyama 930-0194, Japan; ktakaako@med.u-toyama.ac.jp (K.T.); T.Hanaoka.com@outlook.jp (T.H.); yukaberry0822@yahoo.co.jp (Y.H.); aajtpx5830@yahoo.co.jp (Y.A.); iori4869@med.u-toyama.ac.jp (I.M.); shin-ya@nsknet.or.jp (S.K.); taando33@gmail.com (T.A.); haruka52@med.u-toyama.ac.jp (H.F.); tajikazu@med.u-toyama.ac.jp (K.T.); minemura@med.u-toyama.ac.jp (M.M.); taka@med.u-toyama.ac.jp (T.T.)

**Keywords:** EUS-FNB, Franseen needle, histopathological assessment, small pancreatic lesions

## Abstract

Background and aim: During endoscopic ultrasound-guided fine needle aspiration biopsy (EUS-FNB), Franseen needles can help collect sufficient tissue to permit histopathological assessment. However, its efficacy might be limited by the size of the targeted lesion. This study aimed to evaluate the feasibility of histopathological assessment of small solid pancreatic lesions using a 22-gauge Franseen needle during EUS-FNB. Methods: This retrospective study evaluated data from all patients who underwent EUS-FNB using a Franseen needle for solid pancreatic lesions at the University of Toyama Hospital between June 2018 and April 2020. Results: The study included 159 patients who had 152 malignant lesions and 7 benign lesions. The malignant lesions included pancreatic cancers (*n* = 134), neuroendocrine neoplasms (*n* = 15), metastatic tumors (*n* = 2), and a solid pseudopapillary neoplasm (*n* = 1). The diagnostic accuracy of EUS-FNB (combining histology and cytology) was 98.7%. However, the histopathological diagnosis was only confirmed for 64.3% of small lesions (<10 mm), relative to 97.2% for larger lesions. Multivariate analysis also revealed that lesion size of <10 mm predicted a less accurate histopathological diagnosis (odds ratio: 6.97, 95% confidence interval: 1.02–47.67; *p* = 0.041). Further analyses revealed a failed histological diagnosis in 4 patients with lesions of <5 mm in size and accurate diagnoses in 9 out of 10 patients with lesions of 5–10 mm in size. Conclusions: The diagnostic accuracy for small lesions (<10 mm), especially for lesions of <5 mm, based on histological examination alone, was significantly lower than that for others (>10 mm). Furthermore, multivariate analysis revealed that only lesion size was an independent predictor of histopathological diagnosis accuracy.

## 1. Introduction

Endoscopic ultrasound-guided fine needle aspiration (EUS-FNA) is the gold standard technique for diagnosing solid pancreatic masses. The pathological assessment was originally based on cytological findings, which provide 85–97% sensitivity, 88–98% specificity, and 78–96% diagnostic accuracy [1,2,3]. However, histopathological assessments provide more information regarding tissue architecture and immunohistological staining, thus facilitating a more accurate and precise diagnosis than that obtainable with cytological assessment [4]. In addition, histopathological findings, including immunostaining, are essential for diagnosing various diseases, such as lymphoma, autoimmune pancreatitis, and other rare tumors. Large-caliber 19-gauge needles have been used to obtain sufficient biopsy samples for histopathological assessment [5,6,7] although this technique has some technical issues related to the stiff shaft and unsharpened tip. To overcome these issues, third-generation FNA needles, including Franseen needles, were recently developed [8]. Franseen needles have three symmetric heels at the tip and their unique shape helps obtain a sufficient sample for histopathological assessment in most cases, even if the needle is a conventional caliber (22-gauge, 22G). Several studies have indicated that using a Franseen needle during EUS-guided fine needle biopsy (EUS-FNB) provides various benefits [9,10,11], although the feasibility of using Franseen needles might be limited for smaller lesions. Therefore, this study aimed to evaluate the feasibility of histopathological assessment for small pancreatic lesions using a 22G Franseen needle during EUS-FNB.

## 2. Materials and Methods

### 2.1. Patients

This retrospective single-center study was conducted at the University of Toyama Hospital and included all patients who underwent EUS-FNB for solid pancreatic lesions between June 2018 and April 2020. During this period, a 22G Franseen needle (Acquire; Boston Scientific, Marlborough, MA, USA) was routinely used as the first biopsy needle at our institution. The patient and lesion characteristics, final diagnosis, diagnostic ability, and adverse events of EUS-FNB using a 22G Franseen needle were investigated. The severity of adverse events was defined according to the American Society for Gastrointestinal Endoscopy classification [12]. This retrospective study protocol was approved by the institutional review board of our institution (R2020096) on 25 August 2020, and registered in the University Hospital Medical Information Network clinical trials registry (UMIN000041511). All patients had provided informed consent for the EUS-FNB procedure.

### 2.2. Procedural Technique

The EUS was performed as an in-patient procedure using a curved linear echoendoscope (GF-UCT260; Olympus Corporation, Tokyo, Japan) connected to an ultrasound scanning system (EU-ME2; Olympus Corporation, Tokyo, Japan). The patients underwent EUS-FNB under conscious sedation with midazolam, and their vital signs were monitored. Two experienced endosonographers (I.Y. and K.T.) performed the EUS-FNB using a 22G Franseen needle (Acquire; Boston Scientific) in all cases, as previously reported [13]. The lesion was punctured via the stomach or duodenum under guidance from real-time EUS imaging, and color Doppler imaging was used to confirm that the puncture path would not disrupt any major vessels or the main pancreatic duct. The stylet was then removed, a 20 mL syringe was attached to the needle, and 10 mL of negative pressure was applied. Several movements were made within the lesion and the suction was slowly released after the movements were completed. The needle was then withdrawn into the sheath and the entire system was withdrawn from the biopsy channel. The aspirated material was expelled onto glass slides by carefully reinserting the stylet into the needle. All patients were observed for ≥24 h after the procedure.

The specimen was macroscopically evaluated, and the whitish portions (macroscopically visible core) were collected and placed on a small piece of filter paper. The sample was then placed in formalin solution for histological examination and the remaining material was smeared on glass slides for cytological examination. As our institution does not have an on-site pathologist or cytologist, punctures were repeated up to three times until a whitish material was macroscopically observed. If the third puncture was not successful, the lesion was diagnosed based on only the cytological examination. 

### 2.3. Definition

In this study, the diagnosis based on the EUS-FNB specimen was determined using the histological and cytological findings. The “histological diagnosis” only considered the histological findings (without cytology) and included pancreatic cancer, neuroendocrine neoplasm (NEN), metastatic tumor, and solid pseudopapillary neoplasm (SPN). The cytological diagnosis was classified as definite, suspected malignancy (including NEN and SPN), or benign, based on the Bethesda system. Cases judged as “definite” or “suspicious” by cytological diagnosis were defined as malignant. The final diagnosis was based on: (1) definite evidence of malignancy from a surgical specimen, (2) a diagnosis of malignancy based on the EUS-FNB findings and clinical/imaging follow-up compatible with malignant disease, or (3) no evidence of malignancy based on the EUS-FNB findings and clinical/imaging follow-up of ≥6 months.

### 2.4. Endpoints

The primary endpoint was the diagnostic accuracy of EUS-FNB for small pancreatic lesions (diameter of <10 mm). The diagnostic histopathological results of small pancreatic lesions were compared with those of other lesions (>10 mm). We also analyzed factors that were associated with the accuracy of the histopathological diagnosis using EUS-FNB.

### 2.5. Statistical Analyses

Continuous variables were presented as median (range) and categorical variables were presented as number (percentage). Univariate and multivariate analyses were performed to identify the factors influencing the accuracy of the histopathological diagnosis using EUS-FNB. The univariate analyses were performed using the chi-squared test or Fisher’s exact test for categorical variables and the Mann-Whitney U-test for continuous variables. The multivariate analysis was performed using a logistic regression model, and factors with a univariate *p*-value of <0.05 were entered into the multivariate model. All analyses were performed using JMP^®^ software (version 15; SAS Institute, Inc., Cary, NC, USA). 

## 3. Results

During the study period, 159 patients underwent EUS-FNB for pancreatic lesions, using a 22G Franseen needle. The baseline patient and lesion characteristics are shown in Table 1. The patients included 94 men and 65 women, and the median age was 71 years (range: 34–89 years). The median lesion size (largest diameter) was 28.4 mm (range: 4.2–76.2 mm), and 14 lesions were <10 mm. The puncture route was through the stomach in 101 cases, through the D1 segment (duodenal bulb) in 36 cases, and through the D2 segment (second part of the duodenum) in 22 cases. The median number of needle passes was 2 (range: 1–3). No adverse events were associated with the EUS-FNB. The cytological diagnoses were of definite malignancy in 121 cases, suspected malignancy in 28 cases, and benign in 10 cases. The histological diagnoses were of adenocarcinoma in 133 cases, NEN in 10 cases, SPN in 1 case, and no malignancy in 15 cases (Table 2).

**Figure 1:**
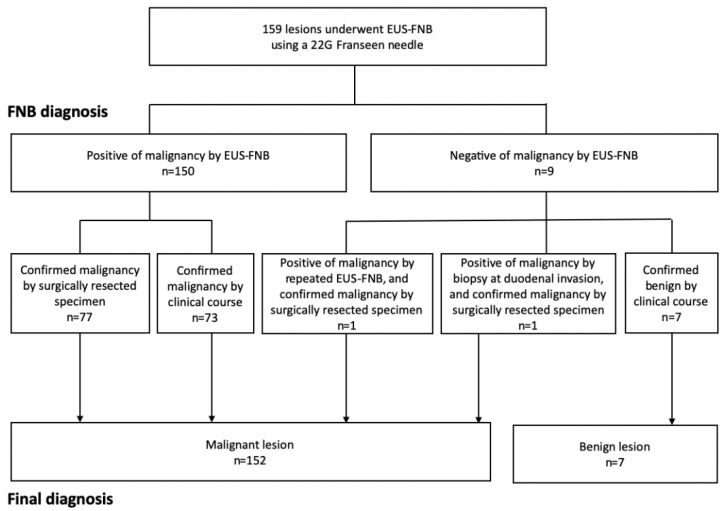
Flowchart of diagnosis via endoscopic ultrasound-guided fine-needle biopsy (EUS-FNB). ERCP, endoscopic retrograde cholangiopancreatography.

The diagnosis based on EUS-FNB was of malignancy in 150 cases and no malignancy in 9 cases. The diagnoses of 150 cases that were considered to have malignancy based on the EUS-FNB were confirmed to be malignancy based on the surgical specimen (77 cases) or based on the clinical follow-up (73 cases). Among the 9 cases that were judged to not be malignant based on the EUS-FNB, 7 cases involved benign lesions based on the clinical follow-up, 1 patient was diagnosed with a malignant lesion after re-examination of the EUS-FNB findings, and 1 patient was diagnosed with duodenal invasion of pancreatic cancer based on findings from endoscopic biopsy and the surgical specimen. Thus, the final diagnoses were of malignant lesions in 152 cases and benign lesions in 7 cases (Figure 1). The malignant lesions included 134 pancreatic cancers, 15 NENs (13 cases with grade 1 NENs and 2 cases with grade 2 NENs), 2 metastatic tumors from lung cancer, and 1 SPN (Table 3).

### 3.1. Diagnostic Results of EUS-FNB

The diagnostic results of EUS-FNB are shown in Table 4. The EUS-FNB procedure (combining histology and cytology) provided 98.7% accuracy, 98.7% sensitivity, 100% specificity, a positive predictive value (PPV) of 100%, and a negative predictive value (NPV) of 77.7%. Based on the histological findings alone, the procedure provided 94.3% accuracy, 94.1% sensitivity, 100% specificity, a PPV of 100%, and an NPV of 40%. The accuracies of the histological findings were 64.3% for cases with lesions of <10 mm and 97.2% for cases with lesions of ≥10 mm (*p* < 0.001) (Table 5). 

### 3.2. Factors Influencing the Accuracy of the Histopathological Diagnosis

Factors influencing the accuracy of the histopathological diagnosis were evaluated using univariate and multivariate analyses (Table 6). Univariate analyses revealed that diagnostic accuracy was significantly associated with age of ≥70 years, lesion size of ≥10 mm, and final diagnosis of pancreatic cancer. However, the diagnostic accuracy was not significantly associated with sex, regular use of antithrombotic drugs, lesion location, serum carbohydrate antigen 19–9 level, or number of needle passes. The three significant variables were included in the multivariate model, which revealed that diagnostic accuracy was only independently associated with lesion size of <10 mm (odds ratio: 6.97, 95% confidence interval: 1.02–47.67, *p* = 0.041).

### 3.3. Subgroup Analysis of Lesions that Were <10 mm

The baseline characteristics and pathological outcomes of cases with lesions that were <10 mm are shown in Table 7. The diagnosis based on EUS-FNB (combining histology and cytology) was accurate in 13 out of 14 cases, although the histopathological diagnosis was only accurate in 9 cases. In particular, a sufficient sample for the histopathological assessment could not be obtained in all 4 cases with lesions of <5 mm, which resulted in inaccurate diagnoses. However, the histopathological diagnoses were accurate in 9 out of 10 cases with lesions measuring in the range of 6–10 mm.

## 4. Discussion

Franseen needles have emerged as a preferable alternative to conventional needles as they can easily obtain larger tissue samples than conventional needles. For example, relative to a conventional needle, a Franseen needle of the same gauge provides approximately 5× the median area of tissue sample for histopathological evaluation [10]. In this context, larger samples preserve tissue architecture and can provide a more accurate and easier pathological diagnosis, relative to smaller samples. We have also reported that a macroscopic on-site evaluation of the macroscopically visible core can help determine the required number of needle punctures, even without rapid on-site cytological evaluation (ROSE) [7], which can reduce the burden on endosonographers and pathologists. Furthermore, obtaining a sufficient core sample can enable genetic analysis and molecular profiling [8,14], which can contribute to personalized treatment selection. Several studies have already confirmed the efficacy of the Franseen needle [15,16,17]. A meta-analysis of 15 studies with 1024 patients revealed that, relative to FNA needles and in the absence of ROSE, FNB needles provided better diagnostic adequacy for solid pancreatic lesions and required fewer needle passes to establish the diagnosis [18]. Our previous study also revealed that use of a Franseen needle provided sufficient tissue samples for histological interpretation in 96% of cases with a single needle pass and in 100% of cases with 3 needle passes [16]. 

Several studies have addressed factors that affect the diagnostic accuracy of EUS-FNA. Uehara et al. [19] reported that the diagnostic accuracy of EUS-FNA was not associated with lesion size, lesion location, or needle size, based on their retrospective study, and concluded that EUS-FNA was useful for diagnosing small pancreatic lesions (<10 mm). However, Agarwal et al. [20] reported that the diagnostic accuracy of EUS-FNA was lower for suspicious pancreatic lesions that were <20 mm, relative to lesions that were >21 mm. Haba et al. [21] also reported that a low diagnostic accuracy was independently associated with a nonpancreatic cancer final diagnosis, pancreatic head lesion location, lesion size of <20 mm, and absence of ROSE. Furthermore, they found that lesion size and ROSE had the greatest influence on diagnostic accuracy. Kurita et al. [22] also reported that the diagnostic accuracy of EUS-FNA was significantly lower for small pancreatic tumors (<5 mm) accompanied by chronic pancreatitis and pancreatic cancer. Thus, small lesion size appears to be associated with lower diagnostic accuracy, although those studies used conventional FNA needles and the pathological diagnosis was mainly based on cytological assessment. Therefore, it is unclear whether EUS-FNB using a Franseen needle is feasible for histopathological assessment of small pancreatic lesions.

The present study revealed that the overall accuracy of EUS-FNB (combining cytology and histology) was 98.7% for all lesions, and the accuracy was still high (94.3%) for lesions that were <10 mm. Nevertheless, the diagnostic accuracy based on the histological examination alone was only 64.3% for small lesions (<10 mm), although the accuracy increased to 97.2% for lesions that were >10 mm (*p* < 0.001). In addition, lower diagnostic accuracy was associated with age <70 years and a non-pancreatic cancer final diagnosis, although these factors were not significant in the multivariate analysis, and only lesion size was an independent predictor of histopathological diagnosis accuracy. This may be related to cases with small lesions (<10 mm) often involving younger patients (<70 years old) and patients with non-NEN tumors (Table 7).

A detailed examination of the EUS-FNB results for lesions that were <10 mm revealed some interesting findings. First, sufficient samples could not be obtained for the histopathological diagnosis in all 4 cases that had lesions of <5 mm although the histopathological diagnosis was accurate in 9 out of 10 cases (90%) that had lesions measuring in the range of 6–10 mm. Thus, a lesion size of <5 mm, rather than <10 mm, may influence the accuracy of the histopathological diagnosis, which may be related to the difficulty of visualizing and puncturing these small lesions. Nevertheless, cytological diagnosis was possible in all 4 cases with lesion sizes of <5 mm, which would suggest that the needle punctured the lesion correctly. Therefore, we conclude that the histopathological diagnosis is likely more difficult than the cytopathological diagnosis in cases with such small lesions. However, combining the cytologic and histopathologic information provided by the biopsy may enhance the diagnostic accuracy in such cases.

Interestingly, all 4 cases with lesions of <5 mm had a cytological diagnosis of NEN, which might have contributed to the failed histopathological diagnosis. However, the diagnostic accuracy of EUS-FNA is generally considered lower for pancreatic cancer than for other pathologies [22]. This may be because pancreatic cancer often involves an intense stromal desmoplastic reaction [23], which may make it difficult to aspirate the sample during FNB and to histopathologically diagnose the limited number of cancer cells in abundant desmoplastic tissue from a small specimen. 

The present study has some limitations. First, the retrospective single-center study design and small number of patients are associated with risks of bias. However, during the study period, we routinely performed EUS-FNB with a Franseen needle for all patients with solid pancreatic masses, regardless of lesion size and location. Nevertheless, large prospective studies are needed to validate our findings. Second, the final diagnoses were confirmed based on the surgical specimen in 79 out of 159 patients, while the EUS-FNB findings and clinical course were used to confirm the diagnoses for the other 80 patients (73 malignant lesions and 7 benign lesions). It is possible that the 7 patients who were diagnosed with benign tumors might actually have had low-grade malignancy, although they did not exhibit any signs of malignancy during a ≥6-month follow-up. Third, the study, including the assessment of diagnostic accuracy, is limited by a very small number of biopsies of lesions of <5 mm.

In conclusion, the diagnostic accuracy for small lesions (<10 mm), especially for lesions of <5 mm, based on the histological examination alone, was significantly lower than for other lesions (>10 mm). Furthermore, multivariate analysis revealed that only lesion size was an independent predictor of histopathological diagnosis accuracy. 

## Figures and Tables

**Table 1:** Baseline characteristics of the patients and the targeted lesions.

	N = 159
Age, years, median (range)	71 (34–89)
Gender (M/F)	94/65
Lesions	
Size, mm, median (range)	28.4 (4.2–76.2)
Size	
<10 mm/10–20 mm/>20 mm	14/36/109
Main location of the lesion	
Head/body/tail	77/44/38

NEN, neuroendocrine neoplasm; SPN, solid pseudopapillary neoplasm.

**Table 2:** Detailed data of endoscopic ultrasound-guided fine needle aspiration biopsy (EUS-FNB).

	N = 159
Puncture route	
Transgastric	101
Trans D1	36
Trans D2	22
Number of needle passes, median (range)	2 (1–3)
FNB diagnosis (cytology)	
Malignant (definitive)	121
Malignant (suspicious)	28
Benign	10
FNB diagnosis (histology)	
Adenocarcinoma	133
NEN	10
SPN	1
No malignancy	15
Complications (EUS-FNB related)	0

NEN, neuroendocrine neoplasm; SPN, solid pseudopapillary neoplasm.

**Table 3:** Final diagnosis of the patients who underwent EUS-FNB.

	N = 159
Pancreatic cancer	134
NEN (NET G1/G2)	15 (13/2)
Metastatic tumor	2 *
SPN	1
Focal chronic pancreatitis	7

NEN, neuroendocrine neoplasm; SPN, solid pseudopapillary neoplasm. * metastasis of lung cancer.

**Table 4:** Diagnostic results of EUS-FNB.

	Accuracy	Sensitivity	Specificity	PPV	NPV
Combined of histology and cytology	98.7%	98.7%	100%	100%	77.7%
Only cytology	94.3%	96.6%	44.4%	97.3%	50.0%
Only histology	94.3%	94.1%	100%	100%	40.0%

PPV, positive predictive value; NPV, negative predictive value.

**Table 5:** Diagnostic histological results of EUS-FNB.

	Accuracy	Sensitivity	Specificity	PPV	NPV
<10 mm	64.3% ^a)^	64.3% ^b)^	N/A ^c)^	100%	0%
≥10 mm	97.2%	98.6%	66.6%	98.6%	66.6%

PPV, positive predictive value; NPV, negative predictive value. ^a)^ *p* < 0.001; ^b)^ *p* < 0.001; ^c)^ Specificity could not be calculated because there were no false-positive and true-negative cases.

**Table 6:** Univariate and multivariate analyses of factors influencing the accurate histopathological diagnosis of EUS-FNB.

Variable	N	Accurate Histopathological Diagnosis (%)	Univariate Analysis			Multivariate Analysis		
			OR	95% CI	*p*-Value	OR	95% CI	*p*-Value
Age (years)								
70≤	97	97.9	6.05	1.21–30.13	0.029	2.56	0.52–12.50	0.251
<70	62	88.7						
Gender								
Female	94	96.8	3.08	0.74–12.81	0.161	—	—	—
Male	65	90.8				—	—	—
Antithrombotic drugs								
+	19	94.7	1.09	0.13–9.24	1.000	—	—	—
-	140	94.3				—	—	—
Location								
Body/tail	82	97.6	4.00	0.80–19.89	0.091	—	—	—
Head	77	91.9				—	—	—
Serum CA19-9 level								
≤37 U/mL	53	90.6	0.37	0.10–1.47	0.161			
>37 U/mL	106	96.2						
Size of lesion								
10 mm<	145	97.2	19.58	4.47–85.80	<0.001	6.97	1.02–47.67	0.041
<10 mm	14	64.3						
Number of needle passes								
1–2	132	94.7	1.43	0.28–7.28	0.650	—	—	—
3	27	92.6				—	—	—
Final diagnosis								
Pancreatic cancer	134	97.8	13.79	3.18–59.80	0.001	3.87	0.53–28.03	0.195
Other diseases	25	76.0						

OR, odds ratio; CI, confidence interval.

**Table 7:** Detailed results of EUS-FNB for lesions smaller than 10 mm.

	Age/Gender	Tumor Size, mm	Tumor Location	Puncture Site	Final Diagnosis	Cytology Result	Histology Result
1	62/F	4.2	Body	Stomach	NET (G1)	Susp. NEN	Inadequate
2	62/F	4.2	Body	Stomach	NET (G1)	Susp. NEN	Inadequate
3	67/F	4.3	Head	D1	NET (G1)	Susp. NEN	Inadequate
4	67/F	4.8	Head	D1	NET (G1)	Susp. NEN	Inadequate
5	86/F	6.5	Head	Stomach	NET (G1)	Benign	Inadequate
6	61/F	6.6	Body	Stomach	NET (G1)	Susp. NEN	NEN
7	67/F	8.5	Head	Stomach	PC	Adenoca.	Adenoca.
8	68/M	8.8	Head	D1	PC	Adenoca.	Adenoca.
9	86/F	8.8	Tail	Stomach	NET (G1)	Susp. NEN	NET (G1)
10	69/F	8.9	Head	Stomach	NET (G1)	Susp. NEN	NET (G1)
11	78/M	9.1	Head	Stomach	PC	Adenoca.	Adenoca.
12	85/M	9.1	Body	Stomach	NET (G1)	Susp. NEN	NEN
13	34/F	9.4	Tail	Stomach	SPN	Susp. SPN	SPN
14	89/M	9.9	Tail	Stomach	NET (G1)	Susp. NEN	NET (G1)

D1, descending part of the duodenum; NET, neuroendocrine tumor; PC, pancreatic cancer; SPN, solid pseudopapillary neoplasm; NEN, neuroendocrine neoplasm; Adenoca., adenocarcinoma; Inadequate, inadequate sample for pathological diagnosis.

## Data Availability

No new data was created or analyzed in this study. Data sharing is not applicable to this article.
